# Student Peer Assessment in Clinical Operative Dentistry: A Retrospective Study

**DOI:** 10.1155/ijod/9268602

**Published:** 2025-11-12

**Authors:** Danya Hashem, Abeer Farag, Amnah A. Algarni, Ranya Zahran Mubarak, Yasser Alwasifi, Somaya Ali Saleh

**Affiliations:** ^1^Department of Restorative Dental Science, College of Dentistry, Taibah University, Madinah, Saudi Arabia; ^2^Department of Restorative Dentistry, College of Dentistry, Deraya University, Minya, Egypt

**Keywords:** cavity preparation, instructor assessment, operative dentistry, peer assessment

## Abstract

**Objectives:**

To compare peer assessment with instructors' assessment of cavity preparations prepared by undergraduate dental students in operative clinical sessions and to assess the effect of different variables such as gender, academic year of the students, and timing on the reliability and accuracy of the peer assessments.

**Methods:**

A retrospective cohort study evaluating 2715 cavities prepared by undergraduate dental students and assessed by instructors and peers using a standardized grading rubric utilized formally within the operative courses. The effect of gender, level of academic year, and timing of assessment was studied. Descriptive statistics and paired sample *t*-test were used to analyze the results.

**Results:**

Significantly higher (*p* < 0.001) peer compared to instructor assessment was found during the first 2 months of the academic year, which gradually reduced towards the end of the academic year. This was consistent between genders and throughout the levels of academic years.

**Conclusion:**

Peer assessment is reliable in gauging the level of understanding and clinical judgment skills of undergraduate dental students. It is recommended to employ peer assessment as a reliable strategy in the active learning process in dental operative clinical courses.

## 1. Introduction

Attaining and maintaining high clinical standards in professional dental practice has increased the need to develop dentists who are self-directed, life-long learners, and reflective practitioners [1]. Achieving the necessary competencies have led to the introduction of different assessment forms [2]. Indeed, assessment is an integral part during the course of education and is usually the main focus for students and the driving force for them to engage in the learning process. Assessment methods can take a multitude of formats and may be classified in many ways [3]. Peer assessment and peer feedback is one form that is increasingly being encouraged to enhance dental students' learning [4]. The learning experience can be enriched by increasing the student's responsibility, inspiring diplomatic criticism, peer integration, and increasing familiarity with evaluation criteria. It also helps overcome unrealistic expectations and encourages reflection, critical skills and lifelong learning. This all leads to an improvement in the student's overall clinical performance [2, 5, 6]. Developing operative skills in restorative dentistry in a clinical environment is a main aspect of training of dental undergraduate students. Determining competence is imperative for students and staff alike and the assessment of clinical skills is important not only for patient safety, but also for informing and providing feedback to the student regarding their performance [6].

Student learning is closely related to methods of assessment and evolving methods are directed towards encouraging deeper learning strategies. Critical thinking and reflective learning is always worth building into the course and the use of peer-group assessment is one means of achieving this [7].

Peer assessment has been defined as an arrangement in which individuals consider the amount, level, value, worth, quality, or success of the products or outcomes of learning of peers of similar status [8]. This promotes deeper learning rather than simply performing a technical skill and has been reported to develop skills in decision making, communication, professionalism, and reflection as reported in a study by Quick [9] in 2016. Although peer assessment has been found to be more valued than used, this suggests that barriers exist in utilizing this mode of assessment. One such barrier may be the potential problems of validity and reliability [6]. Indeed, “friendship” or collusive marking, reluctance to accept any responsibility for assessing or criticizing their friends, and students not accepting peer assessment and peer feedback as accurate and helpful have all been reported to be limitations of peer assessment [2].

However, implementation of peer assessment using specific and standardized criteria with a clear rationale and preknown guidelines for the evaluation of clinical performance has a well-documented benefit and this study will help shed the light on the validity and reliability of this assessment method in the clinical operative field of dentistry which is lacking in the current literature compared to the preclinical operative setting. At Taibah University College of Dentistry, peer assessment has been incorporated as part of the clinical operative dentistry curriculum throughout the year for students in year 4, 5, and 6. Whereas, each cavity preparation and restoration done in the clinical operative session has to be assessed by both a peer and an instructor.

Therefore, the aim of this research project was first to evaluate the reliability and accuracy of peer assessment compared to instructors' assessment of cavity preparations of 4^th^, 5^th^ and 6^th^ year students in operative dentistry clinical sessions. Second, to assess the effect of different variables such as gender, level of academic year of the students, and timing of the assessment on the reliability and accuracy of the peer assessments.

The null hypothesis is that there is no difference between the marks obtained from the peer assessment compared to the marks obtained from the instructors in the assessment of cavity preparations in the operative dentistry clinical sessions at Taibah University College of Dentistry. There is no difference between male and female peer assessment compared to instructor assessment and no difference among the academic levels of the peer assessment and timing of assessment.

## 2. Materials and Methods

### 2.1. Study Design, Participants, and Setting

This is a retrospective cohort study conducted at the Department of Restorative Dental Science at Taibah University College of Dentistry. Two undergraduate students were assigned to practice inside the same clinical cubicle at the same clinical session when large numbers of undergraduate students are enrolled in a specific year. One student was assigned as operator, and his/her peer was assigned as assistant, and they shift their roles the next session. Peer assessment is undertaken as part of the operative course formative assessment at the college, which involves student peer assessment process for each cavity preparation and restoration before assessment by the instructor throughout the year. This is assessed qualitatively according to a definite assigned rubric with predetermined assessment criteria adapted from the USPHS criteria and approved by the operative dentistry department. This rubric has been used for assessment of cavity preparations and restorations in the department for many years [10]. The rubric was explained at the start of each academic year to students and all faculty members participating in the course and they are trained and calibrated on using the rubric before the start of the operative clinical sessions. Each cavity preparation was assessed twice; by the clinical instructor and by a peer using the same rubric. All the past data of this qualitative assessment of the prepared cavity in our archive was used in this study.

### 2.2. Selection of Samples

A total of 2715 prepared cavities performed by 4^th^, 5^th^ and 6^th^ year undergraduate dental students throughout the year were selected in this study according to the following inclusion criteria:

1. Only preparations following Black's classification were selected.
2. Each cavity preparation was assessed qualitatively according to the assigned rubric.
3. Each cavity preparation should be assessed by an instructor with a PhD in Operative dentistry and at least 5 years' teaching experience for undergraduate students.
4. Each cavity preparation should be assessed by a peer of the same academic year using the same assigned rubric, before being assessed by the instructor.

Students were classified into males and females, then, further classified according to the academic year into three groups, 4^th^, 5^th^ and 6^th^ years, respectively. Each group was further classified according to the time of cavity preparation assessment into eight subgroups starting from the first month of the academic year (September) to the last (April). This was done to assess the monthly progress of effectiveness of peer assessment strategy throughout the academic year.

The mean mark obtained by qualitative assessment of prepared cavities by both the instructor and the peer were compared and statistically analyzed at different levels of the study. SPSS V.26 was used for statistical analysis. Paired sample *t*-test was used to compare means with a significance level at *p* < 0.05 and <0.01. Ethical approval for this study was gained by Taibah University College of Dentistry Research Ethics Committee (TUCDREC/20200421/DFHashem).

## 3. Results

**Figure 1:**
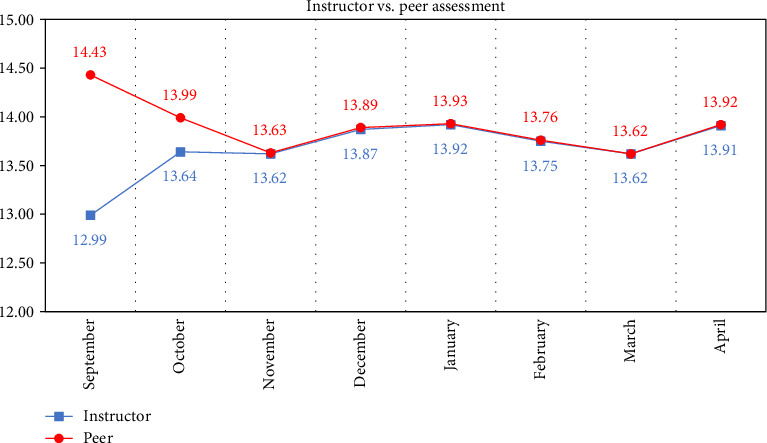
Line graph of the mean mark of the monthly instructor vs. peer assessment.

Descriptive statistics of the marks obtained from the monthly cavity preparation assessment of the instructor compared to peer marks are displayed in Table 1 and Figure 1 from the month of September to April.

Paired samples *t*-test of the monthly mean cavity preparation marks recorded for the first 2 months of the academic year (September and October) displayed significant difference (*p* < 0.001) between instructors and peers, regardless of gender or the academic year. Peer assessment marks were higher than the instructor marks where the mean peer mark was 14.43 compared to 12.99 for instructor in September and 13.99 peer mean mark compared to 13.64 for instructor in October. The difference between the two mean assessment marks were reduced to minimal by the third month—November—till the end of the academic year at April with a statistically non-significant difference, as shown in Table 2.

**Figure 2:**
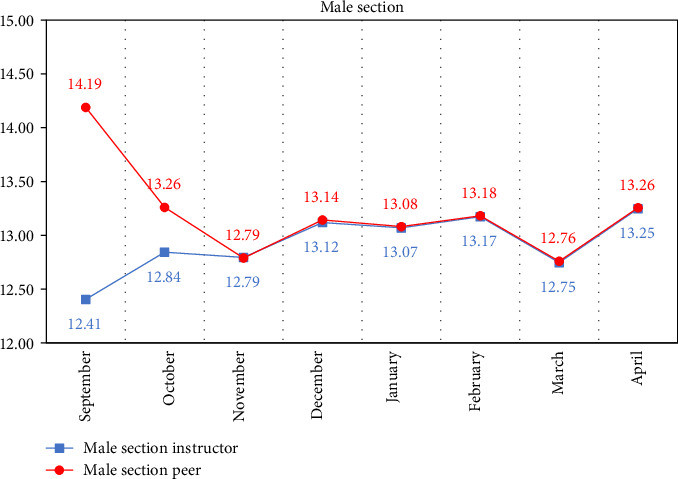
Line graph of the mean mark of the monthly instructor vs. peer assessment of male section.

**Figure 3:**
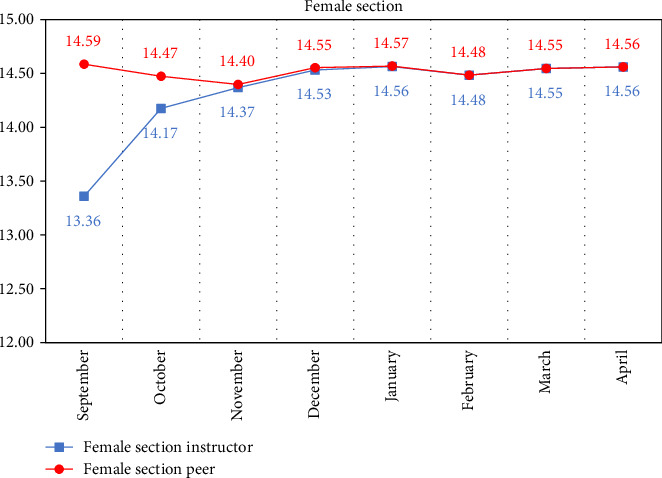
Line graph of the mean mark of the monthly instructor vs. peer assessment of female section.

Descriptive statistics of the marks obtained from the monthly cavity preparation assessment of the instructor compared to peers according to gender are displayed in Table 3 and Figures 2 and 3 from the month of September to April.

Paired samples *t*-test of the monthly mean cavity preparation mark of instructors and peers for each gender also recorded a significant difference at *p* < 0.001 for the first 2 months of the academic year. This difference became non-significant starting from the third month till the end of the year (April). Peer assessment marks for both genders were higher in the first 2 months compared to those of the instructor. For males, the mean peer mark in September was 14.19 compared to 12.41 for the instructor, while for females, the mean peer mark was 14.59 compared to 13.36 for the instructor. In October, the mean peer mark was 13.26 and 14.47 for males and females, respectively, compared to 12.84 and 14.17 for the instructors, as shown in Table 4.

Descriptive statistics of the marks obtained from the monthly cavity preparation assessment of the instructor compared to peers according to academic year are displayed in Table 5 from the month of September to April.

In the first 2 months of the academic year, paired sample *t*-test demonstrated a statistically significant difference (*p* < 0.001) between the marks of the peers and instructors for each academic year with peer marks being higher than the instructors for 4^th^, 5^th^, and 6^th^ year students during September and October. This difference became nonsignificant from November till the end of the year (Tables 6).

## 4. Discussion

The current study investigated the reliability of dental students' skills in assessing their peers' cavity preparation in comparison to instructors in an operative dentistry clinical setting. The rational for introducing peer assessment in the operative undergraduate clinical courses was to promote higher levels of thinking, enhance critical skills, and improve interpersonal skills and reflection. Additionally, a greater understanding of the assessment process and criteria gives a greater appreciation of the requirements of the exercise and thereby improved student's overall clinical performance [6].

The overall results differed according to the assessment time, regardless of gender and academic year. The first 2 months of the academic year (September and October) students seemed to overestimate their peer's performance. This was demonstrated by the significant difference in marks between the peer assessor and the instructor, for both genders. Although the students were introduced and trained on the elements of the assessment rubric, other factors may have contributed to this difference. These factors may include adjusting to the learning environment at the beginning of the academic year as well as the influence of friendship. In agreement with this observation, Khan et al. [11] described the tendency of students to give their peers higher marks in OSCE when compared to faculty. This trend was also found in another study by Alfakhry et al. [12] where students' peer-assessment was significantly higher than faculty when using grading rubrics to assess clinical performance in operative dentistry.

The peer–instructor difference in assessment gradually diminished with time, demonstrated by the decrease in peer mean marks, and the increase in mean marks given by instructors. The gradual increase in instructors' marks indicated improvement in students' psychomotor and clinical performance from the beginning of the academic year as they reach the middle of the semester. While the decrease in the students' mean scores overtime may imply development in students' experience as assessors, as well as improvement in their clinical judgment skills. This observation is in keeping with a previous study by Tricio et al. [2], who conducted a 3-month study on preclinical and clinical peer assessment. After training, both groups were able to discriminate between peer-assessed domains and detected improvement in peers' performance overtime. Moreover, peer scores of both groups showed a positive correlation with their mean end-of-year examination marks. They reported positive perceptions of the peer protocol, but without observable educational impact which might be justified by the short duration of that study [2]. Another study investigated the differences between marks given by a peer group acting as examiner when compared with experienced assessors. In the study, artificial teeth were prepared by preclinical students and were randomly allocated for assessment to one of six student groups who graded each tooth preparation using a list of grade descriptors. The same preparations were again assessed by two experienced restorative academic examiners using the same guidance. The results presented no significant differences in grades given by experienced examiners to those presented by peer groups. Hence, they concluded that peer assessment may be useful to encourage a greater understanding of concepts and principles underlying the development of operative skills [6].

The nonsignificant difference between instructor and peer marks for 4^th^ year students in some occasions could be due to the very low number of cavity preparations done in December and April. Students were reluctant sometimes to participate in evaluating their acquaintances, which could be due to their concerns to criticize their colleagues and affect their friendship. This is one of the known limitations that has been related to peer-assessment [3]. In general, there was fluctuation in 4^th^ year students' peer assessment. As this is the first year of clinical operative dentistry, students may take longer time to adapt to the criteria and improve their clinical judgment skills until reaching reliable assessment skills. For higher academic years such as 5^th^ and 6^th^ years, after 2 months the data became more consistent and reliable thereon. This trend is similar to those found in earlier findings where 2^nd^ year dental students displayed less reliable data compared to 5^th^ year in assessing the preclinical and clinical procedural performance, respectively [2].

The nonsignificant difference in peer and instructor assessments after the first 2 months suggests that both students and instructors applied the rubric criteria with the same level of understanding and fairness. This observation is consistent with previous reports in which dental students showed no significant difference between peers and trainers in assessing clinical skills during tooth extraction [13]. This reveals that criteria used in the rubric were explicit, firmly set and therefore, well understood by both assessors. Thus, the clarity of assessment criteria has been advocated to improve students' judgment of their peers' performance [3].

In our case, peer-assessment was implemented within the students' total grades of clinical operative courses. Therefore, students were well-instructed and trained on the assessment criteria for cavity preparation. In addition, students understood their responsibility in performing accurate and fair assessment. Indeed, students' training and their familiarity with the assessment criteria are crucial factors for the reliability of peer-assessment [14]. This was reflected in the mean values of the results which ranged between 12.5 and 14.5 out of a total of 15 displaying a narrow range which indicates general consistency and that the assessment criteria were stringent enough to limit variability.

The study has a few limitations where there was no assessment of the intra- nor interrater reliability between faculty assessors. However, faculty were trained to assess using the grading rubric for many years which according to previous studies can increase the validity and reliability of the used assessment method [15]. Furthermore, peer-assessment bias and friendship bias cannot be excluded although students are well informed that peer-assessment was a formal part of the evaluation process. Further research is needed extending for a longer period of time and including different institutions. Nevertheless, results obtained from this study support the use of peer assessment to enhance the clinical judgment skills of dental students. Additionally, peer assessment was found to be an effective learning method in the development of other skills as well, such as communication, professionalism, decision making, and reflection [9, 15, 16]. Therefore, incorporation of peer-assessment in different undergraduate dental courses is encouraged [17].

## 5. Conclusions

The null hypothesis stating that there is no difference between the marks obtained from the peer assessment compared to those obtained from the instructors in the assessment of cavity preparations in the operative dentistry clinical sessions was partially accepted. This was affected by the time of peer-assessment where a significant difference was found between the peer and instructor assessment during the beginning of the year which gradually reduced until no difference was found towards the end of the academic year. This was the same for both genders and all levels of academic year. Within the limitations of this study, we conclude that peer assessment is reliable in gauging the level of understanding and clinical judgment skills of undergraduate dental students. It is recommended to employ peer assessment as a reliable strategy in the active learning process in dental operative clinical courses.

## Figures and Tables

**Table 1:** Descriptive statistics of the monthly instructor vs. peer assessment of cavity preparations.

Month	Assessor	Mean mark^a^	Total number of cavity preparations	Std. deviation	Std. error mean
September	Instructor	12.99	287	1.518	0.090
	Peer	14.43	287	0.794	0.047

October	Instructor	13.64	431	1.175	0.057
	Peer	13.99	431	0.974	0.047

November	Instructor	13.62	389	1.292	0.066
	Peer	13.63	389	1.283	0.065

December	Instructor	13.87	89	1.025	0.109
	Peer	13.89	89	1.027	0.109

January	Instructor	13.92	401	1.114	0.056
	Peer	13.93	401	1.107	0.055

February	Instructor	13.75	415	1.034	0.051
	Peer	13.76	415	1.026	0.050

March	Instructor	13.62	474	1.273	0.058
	Peer	13.62	474	1.260	0.058

April	Instructor	13.91	229	1.081	0.071
	Peer	13.92	229	1.063	0.070

^a^The mean marks were out of a total of 15.

**Table 2:** Paired samples *t*-test of the monthly instructor vs. peer assessment of cavity preparations.

Month	Assessor	Paired differences					*t*	df	Sig. (2-tailed)
		Mean	Std. deviation	Std. error mean	95% confidence interval of the difference				
					Lower	Upper			
September	Instructor–peer	−1.443	1.264	0.075	−1.589	−1.296	−19.337	286	0.000^a^
October	Instructor–peer	−0.346	0.737	0.035	−0.415	−0.276	−9.740	430	0.000^a^
November	Instructor–peer	−0.013	0.232	0.012	−0.036	0.010	−1.091	388	0.276
December	Instructor–peer	−0.022	0.336	0.036	−0.093	0.048	−0.630	88	0.530
January	Instructor–peer	−0.007	0.166	0.008	−0.024	0.009	−0.904	400	0.366
February	Instructor–peer	−0.005	0.155	0.008	−0.020	0.010	−0.632	414	0.528
March	Instructor–peer	−0.006	0.166	0.008	−0.021	0.009	−0.832	473	0.406
April	Instructor–peer	−0.004	0.239	0.016	−0.035	0.027	−0.277	228	0.782

^a^Indicates significant values.

**Table 3:** Descriptive statistics of the monthly instructor vs peer assessment of cavity preparation according to gender.

Month	Gender	Assessor	Mean mark	Total number of cavity preparations	Std. deviation	Std. error mean
September	Male	Instructor	12.41	111	1.781	0.169
		Peer	14.19	111	0.977	0.093
	Female	Instructor	13.36	176	1.192	0.090
		Peer	14.59	176	0.608	0.046

October	Male	Instructor	12.84	173	1.188	0.090
		Peer	13.26	173	0.932	0.071
	Female	Instructor	14.17	258	0.806	0.050
		Peer	14.47	258	0.643	0.040

November	Male	Instructor	12.79	185	1.189	0.087
		Peer	12.79	185	1.181	0.087
	Female	Instructor	14.37	204	0.852	0.060
		Peer	14.40	204	0.803	0.056

December	Male	Instructor	13.12	42	0.861	0.133
		Peer	13.14	42	0.872	0.134
	Female	Instructor	14.53	47	0.620	0.090
		Peer	14.55	47	0.619	0.090

January	Male	Instructor	13.07	172	1.012	0.077
		Peer	13.08	172	1.005	0.077
	Female	Instructor	14.56	229	0.670	0.044
		Peer	14.57	229	0.663	0.044

February	Male	Instructor	13.17	231	0.842	0.055
		Peer	13.18	231	0.830	0.055
	Female	Instructor	14.48	184	0.754	0.056
		Peer	14.48	184	0.754	0.056

March	Male	Instructor	12.75	245	1.045	0.067
		Peer	12.76	245	1.026	0.066
	Female	Instructor	14.55	229	0.716	0.047
		Peer	14.55	229	0.716	0.047

April	Male	Instructor	13.25	113	1.040	0.098
		Peer	13.26	113	1.007	0.095
	Female	Instructor	14.56	116	0.636	0.059
		Peer	14.56	116	0.636	0.059

**Table 4:** Paired samples *t*-test of the monthly instructor vs. peer assessment according to gender.

Month	Gender	Assessor	Paired differences							
						95% confidence interval of the difference				
			Mean	Std. deviation	Std. error mean	Lower	Upper	*t*	df	Sig. (2-tailed)
September	Male	Instructor–peer	−1.784	1.480	0.140	−2.062	−1.505	−12.702	110	0.000^a^
	Female	Instructor–peer	−1.227	1.055	0.080	−1.384	−1.070	−15.428	175	0.000^a^

October	Male	Instructor–peer	−0.416	0.869	0.066	−0.547	−0.286	−6.296	172	0.000^a^
	Female	Instructor–peer	−0.298	0.630	0.039	−0.376	−0.221	−7.609	257	0.000^a^

November	Male	Instructor–peer	0.005	0.128	0.009	−0.013	0.024	0.576	184	0.565
	Female	Instructor–peer	−0.029	0.296	0.021	−0.070	0.011	−1.418	203	0.158

December	Male	Instructor–peer	−0.024	0.348	0.054	−0.132	0.085	−0.443	41	0.660
	Female	Instructor–peer	−0.021	0.329	0.048	−0.118	0.075	−0.443	46	0.660

January	Male	Instructor–peer	−0.012	0.152	0.012	−0.035	0.011	−1.000	171	0.319
	Female	Instructor–peer	−0.004	0.175	0.012	−0.027	0.018	−0.377	228	0.706

February	Male	Instructor–peer	−0.009	0.161	0.011	−0.030	0.012	−0.816	230	0.415
	Female	Instructor–peer	0.000	0.148	0.011	−0.022	0.022	0.000	183	1.000

March	Male	Instructor–peer	−0.012	0.192	0.012	−0.036	0.012	−1.000	244	0.318
	Female	Instructor–peer	0.000	0.132	0.009	−0.017	0.017	0.000	228	1.000

April	Male	Instructor–peer	−0.009	0.283	0.027	−0.062	0.044	−0.332	112	0.740
	Female	Instructor–peer	0.000	0.187	0.017	−0.034	0.034	0.000	115	1.000

^a^Indicates significant values.

**Table 5:** Descriptive statistics of the monthly instructor vs. peer assessment according to the academic year.

Month	Academic year	Assessor	Mean mark	Total number of cavity preparations	Std. deviation	Std. error mean
September	4th year	Instructor	13.50	24	1.319	0.269
		Peer	14.83	24	0.381	0.078
	5th year	Instructor	12.86	218	1.577	0.107
		Peer	14.41	218	0.822	0.056
	6th year	Instructor	13.33	45	1.206	0.180
		Peer	14.33	45	0.769	0.115

October	4th year	Instructor	13.00	61	1.506	0.193
		Peer	14.28	61	1.051	0.135
	5th year	Instructor	13.64	310	1.108	0.063
		Peer	13.82	310	0.962	0.055
	6th year	Instructor	14.30	60	0.671	0.087
		Peer	14.55	60	0.622	0.080

November	4th year	Instructor	13.17	75	1.899	0.219
		Peer	13.24	75	1.880	0.217
	5th year	Instructor	13.65	266	1.061	0.065
		Peer	13.65	266	1.061	0.065
	6th year	Instructor	14.15	48	1.072	0.155
		Peer	14.15	48	1.072	0.155

December	4th year	Instructor	13.50	2	0.707	0.500
		Peer	14.00	2	0.000	0.000
	5th year	Instructor	14.06	33	0.747	0.130
		Peer	14.06	33	0.747	0.130
	6th year	Instructor	13.76	54	1.164	0.158
		Peer	13.78	54	1.176	0.160

January	4th year	Instructor	13.96	79	0.649	0.073
		Peer	13.97	79	0.640	0.072
	5th year	Instructor	13.90	187	1.238	0.091
		Peer	13.90	187	1.238	0.091
	6th year	Instructor	13.93	135	1.154	0.099
		Peer	13.95	135	1.135	0.098

February	4th year	Instructor	13.86	135	0.755	0.065
		Peer	13.86	135	0.755	0.065
	5th year	Instructor	13.93	149	1.137	0.093
		Peer	13.93	149	1.137	0.093
	6th year	Instructor	13.44	131	1.097	0.096
		Peer	13.46	131	1.076	0.094

March	4th year	Instructor	12.99	94	1.410	0.145
		Peer	13.00	94	1.376	0.142
	5th year	Instructor	13.50	111	1.285	0.122
		Peer	13.50	111	1.285	0.122
	6th year	Instructor	13.88	269	1.129	0.069
		Peer	13.89	269	1.119	0.068

April	4th year	Instructor	13.29	7	1.976	0.747
		Peer	13.43	7	1.618	0.612
	5th year	Instructor	13.73	33	1.180	0.205
		Peer	13.73	33	1.180	0.205
	6th year	Instructor	13.97	189	1.015	0.074
		Peer	13.97	189	1.015	0.074

**Table 6:** Paired samples *t*-test of the monthly instructor vs. peer assessment according to the academic year.

Month	Academic year	Assessor	Paired differences					*t*	df	Sig. (2-tailed)
			Mean	Std. deviation	Std. error mean	95% confidence interval of the difference				
						Lower	Upper			
September	4th year	Instructor–peer	−1.333	1.239	0.253	−1.857	−0.810	−5.270	23	0.000^a^
	5th year	Instructor–peer	−1.546	1.309	0.089	−1.721	−1.371	−17.432	217	0.000^a^
	6th year	Instructor–peer	−1.000	0.929	0.139	−1.279	−0.721	−7.218	44	0.000^a^

October	4th year	Instructor–peer	−1.279	0.951	0.122	−1.522	−1.035	−10.502	60	0.000^a^
	5th year	Instructor–peer	−0.181	0.557	0.032	−0.243	−0.118	−5.710	309	0.000^a^
	6th year	Instructor –peer	−0.250	0.600	0.077	−0.405	−0.095	−3.227	59	0.002^a^

November	4th year	Instructor–peer	−0.067	0.380	0.044	−0.154	0.021	−1.521	74	0.133
	5th year	Instructor–peer	0.000	0.150	0.009	−0.018	0.018	0.000	265	1.000
	6th year	Instructor–peer	0.000	0.292	0.042	−0.085	0.085	0.000	47	1.000

December	4th year	Instructor–peer	−0.500	0.707	0.500	−6.853	5.853	−1.000	1	0.500
	5th year	Instructor–peer	0.000	0.354	0.062	−0.125	0.125	0.000	32	1.000
	6th year	Instructor–peer	−0.019	0.307	0.042	−0.102	0.065	−0.444	53	0.659

January	4th year	Instructor–peer	−0.013	0.113	0.013	−0.038	0.013	−1.000	78	0.320
	5th year	Instructor–peer	0.000	0.180	0.013	−0.026	0.026	0.000	186	1.000
	6th year	Instructor–peer	−0.015	0.172	0.015	−0.044	0.014	−1.000	134	0.319

February	4th year	Instructor–peer	0.000	0.173	0.015	−0.029	0.029	0.000	134	1.000
	5th year	Instructor–peer	0.000	0.164	0.013	−0.027	0.027	0.000	148	1.000
	6th year	Instructor–peer	−0.015	0.123	0.011	−0.037	0.006	−1.420	130	0.158

March	4th year	Instructor–peer	−0.011	0.103	0.011	−0.032	0.010	−1.000	93	0.320
	5th year	Instructor–peer	0.000	0.302	0.029	−0.057	0.057	0.000	110	1.000
	6th year	Instructor–peer	−0.007	0.086	0.005	−0.018	0.003	−1.417	268	0.158

April	4th year	Instructor–peer	−0.143	0.378	0.143	−0.492	0.207	−1.000	6	0.356
	5th year	Instructor–peer	0.000	0.500	0.087	−0.177	0.177	0.000	32	1.000
	6th year	Instructor–peer	0.000	0.146	0.011	−0.021	0.021	0.000	188	1.000

^a^Indicates significant values.

## Data Availability

The data that support the findings of this study are available from the corresponding author upon reasonable request.
